# Home care aides’ attitudes to training on oral health care

**DOI:** 10.1371/journal.pone.0249021

**Published:** 2021-04-12

**Authors:** Wei-Chung Hsu, Yen-Ping Hsieh, Shou-Jen Lan

**Affiliations:** 1 Department of Radiation Oncology, Chung Kang Branch, Cheng—Ching General Hospital, Taichung, Taiwan; 2 Department of Long-term Care, National Quemoy University, Kinmen, Taiwan; 3 School of Basic Medical Science, Putian University, Putian, China; Centre Hospitalier Regional Universitaire de Tours, FRANCE

## Abstract

This study investigated home care aides’ (HCAs) oral health care experience, knowledge, and their intention to receive professional training, to explain and predict factors of their intention to receive such training. This cross-sectional study collected data through a structured questionnaire. HCAs affiliated with home care agencies in Taichung, Taiwan were recruited through purposive sampling. A total of 487 questionnaires were distributed from September to December 2015 with 280 valid responses collected (57.4%).This study predicted the factors of HCAs’ intention to receive oral health care training through a decision tree analysis. The decision tree model classified the respondents with an accuracy of 77.5%. The optimal predictor variable was oral health care knowledge (χ^2^ = 66.662, *p* < 0.0001). Among the low-scoring respondents on oral health care knowledge, 76.4% were classified in the “uninterested” group, whereas 84.8% of the high scorers were classified in the “interested” group. The second best predictor variable was whether oral health care is part of the job responsibility (χ^2^ = 7.979, *p* = 0.007). Among those who answered Yes, 92.9% were in the interested group, as were 76.5% of those who answered No. It is recommended to add “disease and oral care-related content” and “safety protection, assessment, and usage of oral care tools during practical oral care process” to the oral healthcare training course content for HCAs in order to improve HCAs’ oral healthcare knowledge and oral care skills. These research findings are valuable and may be taken into account in the future development of the in-service educational training of oral healthcare for HCAs.

## Introduction

The concept of aging in place is to encourage older adults to live permanently in a familiar living environment. Appropriate services are crucial to facilitate older people’s safety at home [1]. To implement this concept, home care services for community-dwelling older adults have been developed in Taiwan since 2008 [2]. The number of home care aides (HCAs) in Taiwan has grown from 4,797 in 2009 to 17,625 in 2019, indicating the gradually increased demand for HCAs [3].

HCAs have unique working patterns. They perform one-on-one services at the client’s private home. In Taiwan, HCAs perform home care service tasks, and prices are according to each service. “Basic daily care” comprises a total of 18 items: assistance with turning, transfer, getting in and out of bed, sitting/leaving seats, teeth brushing and face washing, getting dressed and undressed, using the toilet, diaper or sanitary napkin changing, urine bucket emptying, toilet cleaning, colostomy bag cleaning, face and hand washing, beard trimming and shaving, finger- and toenail trimming, medication assistance, bed making (and bed sheet changing), and perineum washing. Customers requiring basic daily care may select services from the aforementioned 18 items according to their needs. The items of basic daily care are priced in 30-minutes units, up to a limit of 3 hours a day [2].

In Taiwan, an HCA serves 2–6 clients daily, moving on to the next client’s home once the service to one client is completed [4]. Existing studies have revealed that HCAs enjoy caring for others and helping their clients, and that they are satisfied with the ability to work independently and have flexible schedules [4, 5].

HCAs service clients with varying physiological and psychological conditions without supervisors or other professionals at the work site to provide immediate support or respond to emergencies. Studies have increasingly explored the possibility of HCA training for various job dilemmas. Topics in these studies encompass occupational safety interventions and health hazards prevention [6, 7], home care for patients with dementia [8] and health promotion training [9, 10]. These training programs have enhanced HCAs’ professional skills and competency.

To our knowledge, there is a dearth of research on oral health care performed by HCAs. Numerous studies have confirmed that older adults’ poor oral health conditions (e.g., tooth loss, dental caries, periodontitis, and dry mouth [11] negatively affect their well-being [12, 13]. In response, existing studies have proposed primary oral health care intervention programs provided collectively by family members, formal or informal HCAs, and dental professionals [14, 15]. However, promoting primary oral health care is challenging in Taiwan because the dental hygienist profession is not well developed. HCAs’ job involve engaging with clients regularly, enabling in-depth understandings of their lifestyles and health conditions [10].

HCAs in Taiwan must be certified by the government under the Home Care Aide Certification Program before taking on the job. The oral care-related content in said Program includes “oral hygiene education” and “helping correctly use tools such as a toothbrush and sponge brush for oral/teeth hygiene, oral submucous, and tongue fur” [16]. This indicates that HCAs in Taiwan possess basic knowledge and skills regarding oral hygiene. Oral care is not a separate service item and belongs under basic daily care; thus, HCAs must perform other types of service and oral hygiene within a limited time. Therefore, this study assessed the possibility of primary oral health care promotion via HCAs.

Although numerous studies have investigated the development and training of care aides in nursing homes on oral care intervention [15, 17], adopting these research outcomes in home care remains questionable. Clients served by HCAs vary substantially in disability levels, particularity, and complexity. In addition, HCAs lack around-the-clock professional support at their work site [4].

Therefore, HCA-specific primary oral health care training must be developed. However, HCAs’ oral health care awareness (e.g., opportunities to engage with clients with oral health care needs, knowledge of oral health care, and willingness to partake in oral health care training) must be understood before developing such training courses or considering their candidacy for primary oral health care promotion.

This study investigated HCAs’ experience and knowledge of oral health care in addition to their expectations of oral health care training. The factors of oral health care training intentions were analyzed through a decision tree model.

## Materials and methods

### Participants

This study conducted the required sample size was calculated using G*Power 3.1 [18]. The minimum sample of 265 participants (effect size = 0.2 with 90% power; alpha = 0.05, two tailed).This study recruited HCAs of home care agencies in Central Taiwan as the participants. As of 2015, 750 HCAs are affiliated with 28 home care agencies in the Taichung area [19]. Among the 18 agencies this study contacted, the supervisors from 7 of them agreed to participate in this study. Upon the supervisors’ consent, this study recruited a purposive sample of HCAs. The research team members orally explained the objectives of this study and the written content of the informed consent to HCAs; the HCAs who agreed to participate were asked to sign an informed consent form. A total of 487 questionnaires were distributed between September and December 2015 with 280 valid responses returned (57.4%).

### Ethics statement

This study was approved by the Institutional Review Board of Cheng—Ching General Hospital in August 2015 (CCGH-NTU-104-002). All work was conducted based on the Declaration of Helsinki ethical principles for research involving human participants.

### Measures

This study referenced relevant past research to compile a structured questionnaire [20–22] divided into HCA demographic data, oral health care experience, oral health care knowledge, and expectations of oral health care training. For content validity, this study invited three experts for review: two home care supervisors familiar with home care business, and a dentist familiar with elder adults’ oral health conditions. This study revised and finalized the questionnaire according to these experts’ advice. For content reliability, the continuous variables were calculated in Cronbach’s α, whereas the dichotomous variables were measured in the Kuder–Richardson Formula.

(1) The six HCA demographic items comprised gender, education, facility type, employment status, HCA certification program, and HCA license.
(2) HCA oral health care experience was measured with 17 true-or-false items. The first 3 items concerned previous encounters with clients’ oral health care needs, whether time was allocated to provide oral health care, and experience with oral health care training courses. The other 14 items concerned encounters with clients having abnormal oral health conditions within the past year. The Kuder–Richardson reliability of these 17 items was 0.5.
(3) The oral health care knowledge scale consisted of 10 items measured with a five-point Likert-type scale (i.e., *strongly disagree*, *disagree*, *neutral*, *agree*, and *strongly agree*), totaling 50 points. A higher score indicates a stronger knowledge of oral health care. The Cronbach’s α of these 10 items is 0.906, and the 10 items are listed as follows:
  1) *The client’s eating or chewing difficulties can be rehabilitated*
  2) *Oral health care improves the client’s overall physical condition*
  3) *Oral health care prevents dental caries and periodontitis*
  4) *Dry mouth increases chewing risks*
  5) *Oral health care prevents respiratory infections and aspiration pneumonia*
  6) *Oral health care mitigates halitosis*
  7) *Oral health care mitigates dry mouth*
  8) *Oral health care improves articulacy*
  9) *Oral health care improves saliva secretion*
  10) *Oral health care improves quality of life*
(4) The scale measuring HCAs’ intention to receive professional training in oral health care consists of 11 items assessed on a five-point Likert-type scale (i.e., *strongly oppose*, *somewhat oppose*, *neutral*, *favor*, and *strongly favor*), totaling 55 points. A higher score indicates a stronger willingness to receive training. The Cronbach’s α of these items is 0.946, and they are listed as follows:
  1) *How to identify the client’s oral health condition*
  2) *How to assess the client’s oral*, *eating*, *and chewing status*
  3) *How to allocate oral health care in a work shift*
  4) *How to adjust between oral health care and other job duties*
  5) *How to ensure proper oral health care instruments*
  6) *How to use oral health care instrument*
  7) *How to use moisturizing gel and mouthwash properly*
  8) *Reviewing*, *purchasing*, *and using oral health care instruments*
  9) *Oral health care methods and knowledge*
  10) *Response to safety hazards on the client*
  11) *HCA’s own safety when providing oral health care*

### Statistical analyses

This study conducted the descriptive and decision tree analysis using SPSS Version 19. We used the skewness and kurtosis of z-score values to check the normality assumption for the continuous variables. HCAs’ age (skewness = −3.42, kurtosis = −0.6) followed a non-normal distribution; thus, the data were processed using nonparametric statistics. Sample data were described using median and interquartile range (IQR); if the observed value was even, the median was the mean of the two medial observed values.

Data mining was performed to uncover useful information and knowledge through statistical and computer techniques. Data mining functions included classification, prediction, affinity group, and clustering. The decision tree analysis predicted the characteristics of HCAs with intention to receive professional training of oral health care.

The decision tree in this study was constructed through Chi-square automatic interaction detection (CHAID), which is suitable for discrete data because it enables the number of branches of an internal node to fluctuate from 3 to the number of groups. The response levels of each predictor are merged in pairs through the chi-square test method to obtain the minimum number of clusters for each predictor level [23]. Consequently, the homogeneous sample units are classified into the same group, and the process of segmentation is completed through repetitive searches. All the paths ranging from the root node to the internal nodes to the leaf node represented the decision rules of classification [24].

In addition, CHAID can handle the classification of continuous and categorical variables. In this study, the dependent variables were dichotomized by median value, whereas the predictors comprised HCA demographics, oral health care experience, and oral health care knowledge scales. CHAID selected the predictor variables with the strongest influence on the dependent variables through chi-square tests in each step. This study set the minimum parent and child node sizes as 100 and 50, respectively. The threshold of eligibility level and merge level were set as *p* < 0.05. The chi-square test method used in this study was the likelihood ratio.

## Results

As shown in Table 1, most of the 280 valid respondents were female and had completed high school, and most of these HCAs were middle-aged (median = 50 y; Q1: 42 y; Q3: 55 y). Most of the HCAs had prior training or were nationally certified, with 90% previously enrolled in HCA certification programs and 70% being licensed. The respondents’ employers were evenly distributed with approximately 50% each in charitable associations and foundations (both nonprofit and in line with the regulations of establishing home care service in Taiwan). The median duration of work experience with their current employer was 46 mo (Q1: 20.25 mo, Q3: 75.75 mo), indicating that most of these HCAs had approximately 4 years of home care experience. The median daily working hours were 7.5 h (Q1: 6 h, Q3: 8 h).

**Table 1:** The characteristics of the home care aide.

Variable	Frequency
	n = 280(%)
Sex	
Male	21 (7.5)
Female	259 (92.5)
Education	
Elementary school	14 (5.0)
Junior high school	61 (21.8)
High school	151 (53.9)
College degree	54 (19.3)
Facilities type	
Charitable associations	141 (50.4)
Foundations	107 (38.2)
Other	32 (11.4)
Employment status	
Official employees	124 (44.3)
Contract Employee	145 (51.8)
Other	11 (3.9)
Home Care Aide Certification Program	
Yes	276 (98.6)
NO	4 (1.4)
Home care Aide License	
Yes	203 (72.5)
NO	77 (27.5)
Age*	50 (Q1:42–Q3:55)
Average working hours in day*	7.5 (Q1:6–Q3:8)
Work experience in current facilities*	46 (Q1:20.25–Q3:75.75)

Note: * was median and quartiles.

46.4% of the respondents provided their clients with oral health care, and 42.9% expressed they did not have time to do so during their shifts. Only 36.1% of the HCAs had received oral health care courses. In the past year, the most commonly reported oral symptoms were poorly fitting dentures, an inability to masticate because of toothache, and chewing difficulty (Table 2).

**Table 2:** HCAs understanding of clients’ oral health care status.

Variable	Participant	Percentage
Whether oral care of clients is within the scope of care?		
Yes	130	46.4
NO	150	53.6
Whether there is time in daily work to provide clients with oral care?		
Yes	160	57.1
NO	120	42.9
Whether courses have been taken to learn about caring for the client’s oral hygiene?		
Yes	101	36.1
NO	179	63.9
In the past year, the most commonly reported clients’ oral symptoms		
Accidentally swallowing teeth	2	0.06
Clients losing their dentures	47	0.07
Client’s dentures falling out or malfunctioning	83	0.12
Dentures hurting the mouth	26	0.04
Fillings falling out	21	0.03
Fingers being bitten	17	0.02
Vomiting during oral health care	13	0.02
Clients refusing oral cleaning	69	0.10
Clients unable to sit properly for a meal	70	0.10
Tooth or gum pain preventing eating	96	0.13
Self-perception of bad breath	48	0.07
Dentures not fitting	103	0.14
White papillae on tongue	29	0.04
Difficulty chewing causing longer mealtimes	93	0.13

Table 3 presents the cross-analysis between “whether the oral care of clients is within the scope of care” and “whether time exists in daily work to provide clients with oral care.” The chi-square test reached statistical significance (p < 0.000). According to the adjusted residual, “HCAs responsible for providing oral care” had less time to provide such care service compared with “HCAs not responsible for providing oral care”.

**Table 3:** The cross-analysis between “whether the oral care of clients is within the scope of care” and “whether time exists in daily work to provide clients with oral care.”

Items		Whether there is time in daily work to provide clients with oral care?		χ^2^
		Yes	No	
Whether oral care of clients is within the scope of care?	Yes	33(-10)	97(10)	99.94
	No	127(10)	23(-10)	
Total		160	120	

Note: The parentheses were adjusted residual values. *p* < 0.0001.

The median score of the oral health care knowledge scale was 39.27 (Q1: 36.51 to Q3: 40.72), indicating moderate awareness among the HCAs. Table 4 presents the median of the items. HCAs had a greater understanding of the first three items, namely “Oral health care mitigates halitosis” (4.07, Q1: 3.44 to Q3: 4.64), “Oral health care prevents dental caries and periodontitis” (4.04, Q1: 3.42 to Q3: 4.63), and “Oral health care improves quality of life” (3.95, Q1: 3.936 to Q3: 4.57).

**Table 4:** The oral health care knowledge scale.

Items	Median	Q1	Q3
The client’s eating or chewing difficulties can be rehabilitated	3.61	3.04	4.26
Oral health care improves the client’s overall physical condition	3.88	3.30	4.51
Oral health care prevents dental caries and periodontitis	4.04	3.42	4.63
Dry mouth increases chewing risks	3.94	3.33	4.57
Oral health care prevents respiratory infections and aspiration pneumonia	3.84	3.25	4.49
Oral health care mitigates halitosis	4.07	3.44	4.64
Oral health care mitigates dry mouth	3.85	3.27	4.49
Oral health care improves articulacy	3.71	3.15	4.38
Oral health care improves saliva secretion	3.82	3.26	4.45
Oral health care improves quality of life	3.96	3.36	4.57

Note: Q1 was quartile 1. Q3 was quartile 3.

The median score for intention to receive professional training in oral health care was 43.12 (Q1: 38.43 to Q3: 44.5), indicating a strong desire to receive professional training. Results of the Friedman test indicated that the preferred training items reached statistical significance (χ^2^(10) = 241.29, *p* < 0.0001). Table 5 presents the median of each item; the first three items, namely “HCA’s own safety when providing oral health care” (3.96, Q1: 3.34 to Q3: 4.58), “Oral health care methods and knowledge” (3.90, Q1: 3.31 to Q3: 4.3), and “Response to safety hazards of the client” (3.85, Q1: 3.27 to Q3: 4.49) were the training items the HCAs preferred.

**Table 5:** HCAs’ intention to receive professional training in oral health care.

Items	Median	Q1	Q3
How to identify the client’s oral health condition	3.73	3.17	4.39
How to assess the client’s oral, eating, and chewing status	3.78	3.22	4.43
How to allocate oral health care in a work shift	3.59	3.03	4.23
How to adjust between oral health care and other job duties	3.57	3.03	4.20
How to ensure proper oral health care instruments	3.82	3.27	4.46
How to use oral health care instrument	3.82	3.26	4.45
How to use moisturizing gel and mouthwash properly	3.82	3.26	4.47
Reviewing, purchasing, and using oral health care instruments	3.74	3.17	4.40
Oral health care methods and knowledge	3.90	3.31	4.53
Response to safety hazards on the client	3.85	3.27	4.49
HCA’s own safety when providing oral health care	3.96	3.34	4.58

Note: Q1 was quartile 1. Q3 was quartile 3. Friedman test:χ2(10) = 241.29, *p* < 0.0001).

To facilitate the decision tree analysis, the skewness and kurtosis of z-score values were used to check the normality assumption for the intention to receive professional training in oral health care (skewness = −2.06, kurtosis = 1.98), which revealed it followed a non-normal distribution. Thus, the HCAs were divided into two groups according to the median score of 43 for intention to receive professional training in oral health care.

**Fig 1:**
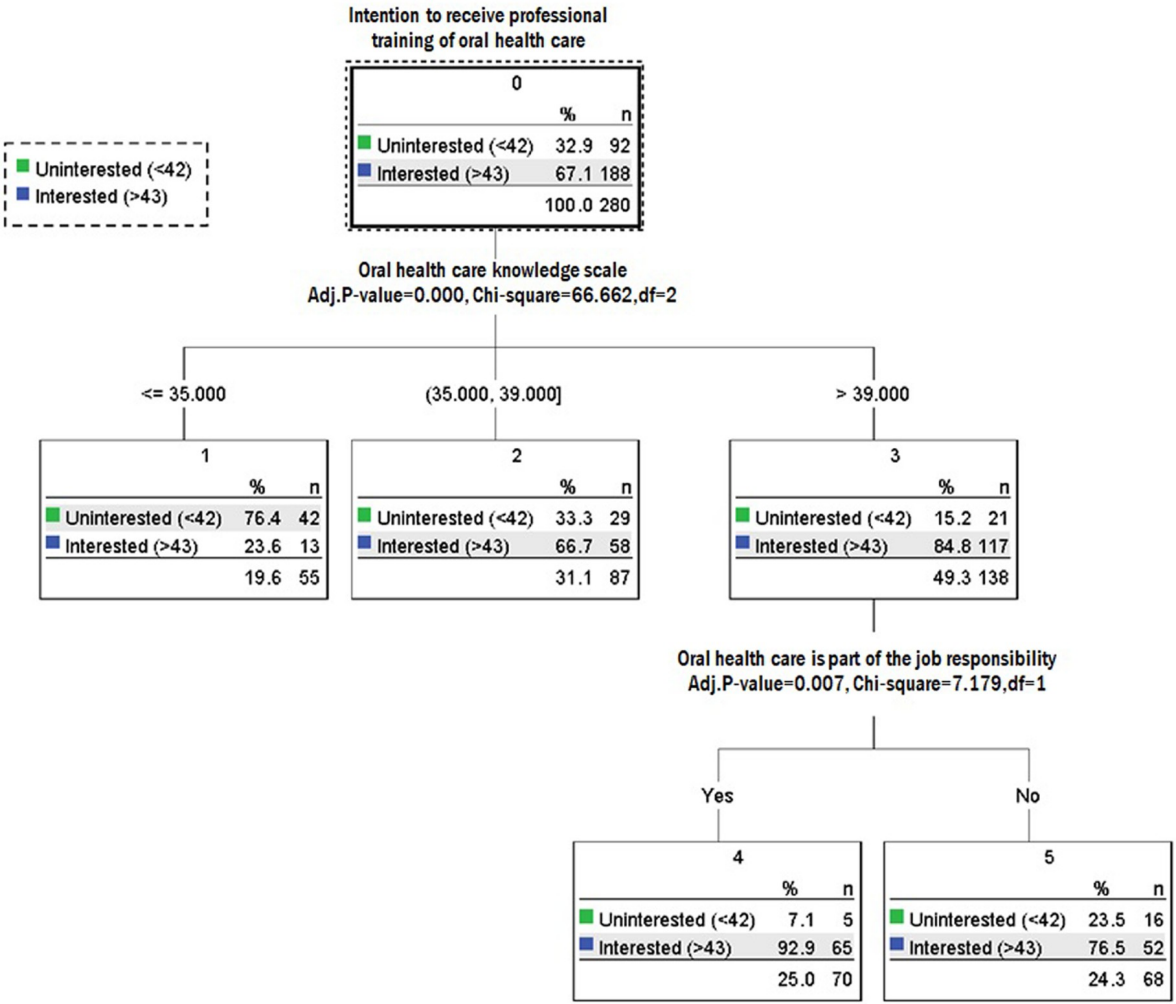
Tree created by the CHAID mode.

The 188 respondents with a score >43 were designated the “interested” group, whereas the other 92 with a score of ≤43 were designated the “uninterested” group. As shown in Fig 1, the score of the oral health care knowledge scale was set as the predictor variable (χ^2^ = 66.662, *p* < 0.0001). Among the low-scoring HCAs (<35), 76.4% were in the uninterested group, 66.7% of those scoring 35–39 were in the interested group, and 84.8% of those scoring >39 were in the desired group.

The second best predictor variable of oral health care knowledge was *whether oral health care is part of the job responsibility* (χ^2^ = 7.179, *p* = 0.007). Among those who answered *Yes*, 92.9% were in the interested group, as well as 76.5% of those who answered *No*. The decision tree model had a 77.5% accuracy of classifying the HCAs’ intention to receive oral health care training.

## Discussion

The results of this study revealed that HCAs whose job contained oral hygiene did not have time to perform it. In addition, the decision tree analysis results demonstrated that the factors that predicted HCAs’ intention to receive oral health care training were *oral health care knowledge* and *whether oral health care is part of the job responsibility*.

Even though oral hygiene is within the HCAs’ job range, the proportion of those who had no time to perform it was high. For HCAs, performing oral hygiene is among the items of basic daily care. Thus, complete oral hygiene care is difficult to accomplish within the designated service time. The oral hygiene process includes the steps of cleaning, using a whole set of cleaning tools, evaluating, and coping with customers’ emergencies, indicating that energy and time are required to provide oral care [25, 26]. Relevant studies have mostly explored the state of teeth brushing in long-term care institutions, and revealed that the time spent by residents on brushing their teeth ranged from 49 seconds [27] to 60.33 seconds (SD = 35.15) [26], indicating that the time for teeth brushing is short. However, if oral hygiene was regarded as a separate care service item, and a payment system was adopted, the oral hygiene process would be more complete but more time-consuming. Among studies on the time for oral hygiene with help from certified nursing assistants (CNAs) who have received oral hygiene training, research in the US indicated that such time lasted approximately 1 minute 20 seconds [27]; research in Japan indicated that it lasted from 1 minute 33 seconds to 3 minutes 59 seconds [28]. Research in the US revealed that such time increased from 3.5 to 6.7 minutes after training [25]. Regarding the time for oral care in long-term care institutions employing professional dental hygienists, research results in Japan indicated that it lasted from 3 minutes 57 seconds to 15 minutes 52 seconds [28], whereas research in Germany found that 36.3 minutes were required (±10.9 min) [29]. The aforementioned information indicates that performing complete oral care requires professionalism, time, and money.

Therefore, it is difficult to continue and conduct oral care tasks without a budget or organizational support [30]. Interventions through public policy are suggested [31]. In 2018, the Long-Term Care Advisory Committee of Taiwan’s Ministry of Health and Welfare proposed the addition of “oral care” to the home care service items in the future [32], indicating that establishing service items exclusively for oral care would be the future trend and would help ensure the implementation of a pay of charge and comprehensive oral care plan; thus, HCAs’ concern over the failure to provide comprehensive oral care due to limited time will be addressed.

Following such a trend, decision tree analysis results of this study helped the researchers to identify two types of trainees willing to receive oral care training, namely HCAs with adequate knowledge about oral health care and those whose actual tasks involve providing oral care. Studies have revealed that the abilities of HCAs receiving oral care training were improved in oral hygiene, oral health care knowledge, and oral evaluation [33], and their willingness to provide oral care service increased [34]. Moreover, the results of this study revealed that HCAs with adequate oral health care knowledge were more willing to participate in oral health care professional training when they performed oral care services.

Oral health care knowledge could be improved through on-the-job training, because by law HCAs in Taiwan must have 20 hours of continuing professional development training each year [16]. Consequently, on-the-job training is prevalent among HCAs [35]. However, studies have stated that formality, repetitiveness, and lack of coherence in training material tend to hinder the effectiveness of these training courses [35, 36]. Another study reported that HCAs express an interest in practical and case-study training courses [35], indicating the gap between available training courses and HCAs’ expectations.

Such a gap could be filled with the results of this study because the oral hygiene knowledge that HCAs knew well was revealed to be basic knowledge on oral health care regarding the prevention of bad breath, tooth decay, and periodontal diseases. Yet, knowledge regarding other diseases of the client induced by inadequate oral care (e.g., respiratory diseases, aspiration pneumonia, oral dryness, and effects on overall health) was somewhat insufficient. This study suggests including oral care-related content in on-the-job training, and incorporating knowledge related to oral and other diseases to enhance HCAs’ oral care knowledge.

The second prediction of the decision tree was that HCAs performing oral care service were more willing to participate in oral health care professional training. The research results revealed that the training course most preferred by HCAs was on how to protect their own and their clients’ safety during oral care, which indicated that HCAs actually performing oral care were more strongly motivated to learn protective measures for the professional oral care process. Moreover, the HCAs were more willing to receive training on the use of oral care-related tools (e.g., toothbrush, mouthwash, and moisturizing gel), and evaluate the status of their client’s mouth, their swallowing function, and their dietary intake, which indicated that the training course preferred by HCAs involved common problems encountered during the oral care process. Thus, this study suggests incorporating tools and evaluation methods of oral care [37, 38] to meet HCAs’ needs regarding actual oral care tasks.

Several limitations exist in this study. First, the purposive sampling method may exhibit sampling errors limiting the inference to a wider population. Second, because the participating HCAs served a wide range of clients, this study could not completely grasp all clients’ oral health conditions. Consequently, the findings may not precisely reflect the difference between the HCAs’ and clients’ oral health care needs.

## Conclusion

Because the oral care service of home care service in Taiwan is combined with other basic daily care services, HCAs usually do not have sufficient time to implement oral healthcare service. It is recommended to independently set the “oral care service” item in the future to enable HCAs to provide oral healthcare service with a fixed time. For the factors affecting the oral healthcare training of HCAs, a simple decision-making model with 77.5% accuracy revealed that HCAs’ oral healthcare knowledge and experience must be accounted for when designing HCA-specific professional training. In the future, it is recommended to add disease and oral care-related oral healthcare knowledge to the oral healthcare training course content for the HCAs and provide safety protection, assessment, and usage of oral care tools during the oral care process to improve HCAs’ knowledge and skills for providing oral care services. These research findings are valuable and may be taken into account in the future development of the in-service educational training of oral healthcare for HCAs.

## Supporting information

S1 File Questionnaires administered to respondent _ Chinese version. (DOC)

S2 File Questionnaires administered to respondent _ English version. (DOC)

S1 Data Relevant data are within the manuscript. (XLSX)
