# Supplementary material for: Home care aides’ attitudes to training on oral health care
Source: PLoS One. 2021 Apr 12;16(4):e0249021. doi: 10.1371/journal.pone.0249021 (PMC8041167; doi:10.1371/journal.pone.0249021)
Supplement: S1 File — (DOC) [file pone.0249021.s001.doc]

**居家服務員的口腔照顧狀況及培訓意願**

**壹、 個人基本資料**

**1. 您的性別： 1) □ 男性 2) □ 女性**

**2. 您的年齡_________歲**

**3. 教育程度： 1) □ 國小以下 2) □ 國中 3) □ 高中 4)□ 專科以上**

**4. 請問您現在工作單位成立類型為何? (請在適合項目打🗹)**

**1) □ 社團法人 2) □ 基金會 3) □ 其他___________________**

**5. 您的工作型態： 1) □ 正式職員 2) □ 約聘僱人員 3) □ 其他:____**

**6. 您是否具有照顧服務員結業證書? 1) □ 有 2) □ 沒有**

**7. 您是否具有勞動部頒發的照顧服務員證照? 1) □ 有 2) □ 沒有**

**8.請問您在現在單位共工作___________________年____________月**

**8. 請問您每日工作約_______________小時**

**貳、案主的口腔照顧工作**

**1.請問您平日服務案主時，案主的口腔照顧是否為照顧範圍之一?**

**1) □ 是 2) □ 不是**

**2.. 請問您平日服務案主時，是否有時間提供案主口腔照顧服務?**

**1) □ 是 2) □ 不是**

**3.請問您是否有上課或學習怎麼照顧案主的口腔清潔?**

**1) □ 沒有 2) □ 有**

**4. 請問過去一年中，您服務的案主是否有發生以下的事情? (可以複數回應，請在適當項目打🗹)**

**1) □ 誤吞牙齒或牙齒的填充物 2) □ 假牙遺失 3) □ 口腔內的假牙掉落或壞掉**

**4) □ 被不適合假牙割傷口腔黏膜 5) □ 幫忙案主口腔清潔時，案主的牙齒或牙齒填充物掉落**

**6) □ 幫忙案主口腔清潔時，被案主咬手指 7) □ 幫忙案主口腔清潔時，案主會想或曾經嘔吐過**

**8) □ 案主拒絕進行口腔清潔 9) □ 案主的用餐時無法正常坐姿**

**10)□ 牙齒或牙齦疼痛無法進食 11) □ 自覺口臭 12) □ 假牙不合**

**13) □ 舌頭有白色的苔 14) □ 咀嚼困難導致進食時間變長**

**參、口腔照顧的知識(請在適當的數字畫○)**

| **題目** | **非常不同意** | **不同意** | **普通** | **同意** | **非常同意** |
| --- | --- | --- | --- | --- | --- |
| **1.您認為案主的進食困難或吞嚥困難是可以復健的嗎?** | **1** | **2** | **3** | **4** | **5** |
| **2.您認為提供口腔照顧，對案主的全身狀況有幫助嗎?** | **1** | **2** | **3** | **4** | **5** |
| **3.您認為提供口腔照顧，對預防蛀牙或牙周病有幫助嗎?** | **1** | **2** | **3** | **4** | **5** |
| **4.您是否同意，口腔乾燥會提升吞嚥的危險?** | **1** | **2** | **3** | **4** | **5** |
| **5.您認為提供口腔照顧，對預防呼吸道感染或吸入性肺炎有幫助嗎?** | **1** | **2** | **3** | **4** | **5** |
| **6.您認為提供口腔照顧，對改善或預防案主口臭有幫助嗎?** | **1** | **2** | **3** | **4** | **5** |
| **7.您認為提供口腔照顧，對減緩口腔乾燥感有幫助嗎?** | **1** | **2** | **3** | **4** | **5** |
| **8.您認為提供口腔照顧，對案主的說話能力有幫助嗎?** | **1** | **2** | **3** | **4** | **5** |
| **9.您認為提供口腔照顧，促進案主的唾液分泌有幫助嗎?** | **1** | **2** | **3** | **4** | **5** |
| **10.您認為提供口腔照顧，對提升案主的生活品質有幫助嗎?** | **1** | **2** | **3** | **4** | **5** |

**肆、請問您希望得到下列何種口腔照顧內容的培訓內容 (請在適當數字打○)**

| **題 目** | **非常不同意** | **不同意** | **普通** | **同意** | **非常同意** |
| --- | --- | --- | --- | --- | --- |
| **1.學習如何掌握案主的口腔狀況** | **1** | **2** | **3** | **4** | **5** |
| **2.學習如何評估案主的口腔、吞嚥及進食狀況** | **1** | **2** | **3** | **4** | **5** |
| **3.如何將口腔照顧放入工作時間** | **1** | **2** | **3** | **4** | **5** |
| **4.口腔照顧工作與其他照顧工作如何相互調整?** | **1** | **2** | **3** | **4** | **5** |
| **5.確保口腔照顧的工具(如牙刷，漱口水等)** | **1** | **2** | **3** | **4** | **5** |
| **7.學習口腔照顧工具的用法** | **1** | **2** | **3** | **4** | **5** |
| **8. 學習口腔保濕凝膠或漱口水的正確用法** | **1** | **2** | **3** | **4** | **5** |
| **9. 諮詢有關檢討、購買或適用口腔照顧工具** | **1** | **2** | **3** | **4** | **5** |
| **10. 學習口腔照顧的方法及知識** | **1** | **2** | **3** | **4** | **5** |
| **11.提供口腔照顧時，案主發生身心危險時的對應方式** | **1** | **2** | **3** | **4** | **5** |
| **12.提供口腔照顧時，如何保護自身安全(例如:不會被咬到等)** | **1** | **2** | **3** | **4** | **5** |
