# Supplementary material for: Home care aides’ attitudes to training on oral health care
Source: PLoS One. 2021 Apr 12;16(4):e0249021. doi: 10.1371/journal.pone.0249021 (PMC8041167; doi:10.1371/journal.pone.0249021)
Supplement: S2 File — (DOC) [file pone.0249021.s002.doc]

Oral care status and training willingness of Home care Aide

Ⅰ The Characteristics of the Home care Aide

1. Sex： 1) □ Male 2) □ Female

2. Age： _________ years old

3. Education： 1) □ Elementary School 2) □ Junior High School 3) □ High School 4)□ College Degree

4. What type of institution was the one you work for when it was founded?

1) □ Charitable 2) □ Foundations 3) □ Other___________________

5. Your employment status： 1) □ Official Employees 2) □ Contract Employee 3) □ Other:____

6. Do you have a certificate of completion for Home Care Aide providers? 1) □ Yes 2) □ No

7. Do you have Home care Aide License ? 1) □ 2) □ No

8. How long have you worked in your current unit?_________________ years ___________ mouths

8. You work approximately _______________ hours per day

Ⅱ、The client’s oral care task.

1. Whether oral care of clients is within the scope of care?

1) □ Yes 2) □ No

2.. Whether there is time in daily work to provide clients with oral care?

1) □ Yes 2) □ No

3. Whether courses have been taken to learn about caring for the client’s oral hygiene?

1) □ Yes 2) □ No

4. In the past year, the most commonly reported clients' oral symptoms： (Multiple Choice Questions. Please tick the appropriate box(s).)

1) □ Accidentally swallowing teeth 2) □ Clients losing their dentures

3) □ Client's dentures falling out or malfunctioning 4) □ Dentures hurting the mouth

5) □ Fillings falling out 6) □ Fingers being bitten 7) □ Vomiting during oral health care

8) □ Clients refusing oral cleaning 9) □ Clients unable to sit properly for a meal

10)□ Tooth or gum pain preventing eating 11) □ Self-perception of bad breath

12) □ Dentures not fitting 13) □ White papillae on tongue

14) □ Difficulty chewing causing longer mealtimes

Ⅲ、The oral health care knowledge scale (Please circle the proper number.)

| Items | Strongly Disagree | Disagree | Neutral | Agree | Strongly Agree |
| --- | --- | --- | --- | --- | --- |
| 1. The client’s eating or chewing difficulties can be rehabilitated | 1 | 2 | 3 | 4 | 5 |
| 2. Oral health care improves the client’s overall physical condition | 1 | 2 | 3 | 4 | 5 |
| 3. Oral health care prevents dental caries and periodontitis | 1 | 2 | 3 | 4 | 5 |
| 4. Dry mouth increases chewing risks | 1 | 2 | 3 | 4 | 5 |
| 5. Oral health care prevents respiratory infections and aspiration pneumonia | 1 | 2 | 3 | 4 | 5 |
| 6. Oral health care mitigates halitosis | 1 | 2 | 3 | 4 | 5 |
| 7. Oral health care mitigates dry mouth | 1 | 2 | 3 | 4 | 5 |
| 8. Oral health care improves articulacy | 1 | 2 | 3 | 4 | 5 |
| 9. Oral health care improves saliva secretion | 1 | 2 | 3 | 4 | 5 |
| 10. Oral health care improves quality of life | 1 | 2 | 3 | 4 | 5 |

Ⅳ、The oral care situation and willingness for training of the home care service provider. Please circle the proper number.

| Items | Strongly Disagree | Disagree | Neutral | Agree | Strongly Agree |
| --- | --- | --- | --- | --- | --- |
| 1. How to identify the client’s oral health condition | 1 | 2 | 3 | 4 | 5 |
| 2.How to assess the client’s oral, eating, and chewing status | 1 | 2 | 3 | 4 | 5 |
| 3.How to allocate oral health care in a work shift | 1 | 2 | 3 | 4 | 5 |
| 4.How to adjust between oral health care and other job duties | 1 | 2 | 3 | 4 | 5 |
| 5.How to ensure proper oral health care instruments | 1 | 2 | 3 | 4 | 5 |
| 7.How to use oral health care instrument | 1 | 2 | 3 | 4 | 5 |
| 8. How to use moisturizing gel and mouthwash properly | 1 | 2 | 3 | 4 | 5 |
| 9. Reviewing, purchasing, and using oral health care instruments | 1 | 2 | 3 | 4 | 5 |
| 10. Oral health care methods and knowledge | 1 | 2 | 3 | 4 | 5 |
| 11. Response to safety hazards on the client | 1 | 2 | 3 | 4 | 5 |
| 12. HCA’s own safety when providing oral health care | 1 | 2 | 3 | 4 | 5 |
